# The secreted FoAPY1 peptidase promotes *Fusarium oxysporum* invasion

**DOI:** 10.3389/fmicb.2022.1040302

**Published:** 2022-10-19

**Authors:** Hengwei Qian, Limin Song, Lulu Wang, Baoshan Wang, Wenxing Liang

**Affiliations:** ^1^College of Life Sciences, Shandong Normal University, Jinan, China; ^2^Key Lab of Integrated Crop Pest Management of Shandong Province, College of Plant Health and Medicine, Qingdao Agricultural University, Qingdao, China

**Keywords:** *Fusarium oxysporum*, secretion, peptidase, virulence, proteomics

## Abstract

The secretion of peptidases from several pathogens has been reported, but the biological function of these proteins in plant-pathogen interactions is poorly understood. *Fusarium oxysporum*, a soil-borne plant pathogenic fungus that causes *Fusarium* wilt in its host, can secrete proteins into host plant cells during the infection process to interfere with the host plant defense response and promote disease occurrence. In this study, we identified a peptidase, FoAPY1, that could be secreted from *F. oxysporum* depending on the N-terminal signal peptide of the protein. FoAPY1 belongs to the peptidase M28 family and exerts peptidase activity *in vitro*. Furthermore, the *FoAYP1* gene knockout strain (∆FoAYP1) presented reduced virulence to tomato plants, but its mycelial growth and conidiation were unchanged. Moreover, FoAYP1 overexpression tomato seedlings exhibited enhanced susceptibility to *F. oxysporum* and *Botrytis cinerea* strains. These data demonstrated that FoAYP1 contributes to the virulence of *F. oxysporum* may through peptidase activity against host plant proteins.

## Introduction

Epidermal cells are the first sites of plant-pathogen interactions, and pathogens can infect the epidermal cells of host plants and expand through hyphae, causing further disease development (Jones and Dangl, 2006). The host cell stimulates plant immune reactions to prevent invading pathogens. To attenuate the plant defense response, pathogens can secrete certain proteins to dictate plant-pathogen interactions and to effectively promote infection of host plant tissues (Akira et al., 2006; Krause et al., 2013; Liu et al., 2021). Depending on their protein properties and functions, the secreted proteins from pathogens can play important roles in several physiological processes. Such proteins are secreted from bacterial pathogens to the host through the type III secretion system (Choi et al., 2017), and pathogenic oomycetes that contain RxLR, CRN or ChxC amino acid sequence motifs deliver these secreted proteins to plant cells (Yang et al., 2017). However, most secreted proteins of fungal pathogens have no conserved motif or system (Yang et al., 2021).

Proteins secreted from pathogens can be divided into two classes according to their functions: enzymatic functional proteins and nonenzymatic functional proteins. Cell wall-degrading enzymes (CWDEs), cutinases, proteases, peptide hydrolases and ribonucleases can be secreted from different pathogens and are enzymatic functional proteins. For instance, FoEG1, which is a GH12 protein secreted from *Fusarium oxysporum* and exerts cellulase activity, is essential for fungal virulence *via* its enzymatic activity (Zhang L. et al., 2021). VdEG1 is another GH12 protein secreted by *Verticillium dahliae* and it plays an important role in virulence (Gui et al., 2017). In addition, FoRnt2, a secreted ribonuclease T2 protein, contributes to the full virulence of *F. oxysporum* in tomato plants *via* its RNase activity (Qian et al., 2022). Moreover, by targeting the host cell nucleus, the secreted Vd424Y protein from *V. dahliae*, a glycoside hydrolase family 11 protein, is required for full virulence (Liu et al., 2021). Another class is comprised of many proteins that have low molecular weight, are rich in cysteine and lack known enzyme activity (Hacquard et al., 2012; Lyu et al., 2016). SsSSVP1 is a secreted protein of *Sclerotinia sclerotiorum* that lacks any known domains and can manipulate plant metabolism for further infection (Lyu et al., 2016).

Secreted peptidases from pathogens also play important roles in host–pathogen interactions. Peptidases can degrade host proteins into amino acids for nutrient acquisition or catabolic activities (Lowe et al., 2015). However, there are other instances of peptidase function in host plant-pathogen interactions through other processes. HopN1, a secreted protein from the bacterium *Pseudomonas syringae*, is a cysteine peptidase that cleaves the host PsbQ protein, which is a key photosynthesis enzyme, to block programmed cell death (Rodríguez-Herva et al., 2012). Furthermore, the Avi-pita protein of *Magnaporthe oryzae* shares a similar sequence with metallopeptidases and interacts with the rice protein Pi-ta, further inducing the defense response in the host plant (Jia et al., 2000). In addition to studying the interaction between the secreted peptidases of pathogens and their hosts, some peptidases from microorganisms have also been used in the food industry, such as leucine aminopeptidase rLap1 (Huang et al., 2015) of *Aspergillus sojae* and lysine aminopeptidase from yeast *Kluyveromyces marxianus* (Ramirez-Zavala et al., 2004), which could increase the degree of hydrolysis protein.

*F. oxysporum* is a soil-borne vascular fungal pathogen that infects host plants through their roots, colonizes xylem vessels and causes root rot and wilting in more than 150 different plant species, such as tomato, banana, melon and pine. *F. oxysporum* has different formae speciales such that they infect only one or a few host species (Sun et al., 2022). The epidemic diseases that ensue are difficult to control because *F. oxysporum* can exist in soil and produce chlamydospores to overcome various conditions. To effectively control this fungus, various chemical fungicides have been used, but these approaches have resulted in strong resistance of *F. oxysporum* to fungicides in the field. Some secreted proteins of *F. oxysporum* have been shown to play an important role in plant-pathogen interactions; however, only a portion of them have been characterized. For instance, some secreted-in-xylem (six) proteins from *F. oxysporum* are required for tomato infection (Jangir et al., 2021; Jenkins et al., 2021). Furthermore, the novel secreted protein FoCP1 has been shown to be important in the virulence of *F. oxysporum* and triggers the immune response of host plants (Li et al., 2019; Liu et al., 2019). In addition, the secreted metalloprotease FoMep1 is involved in full virulence against tomato because it can reduce the antifungal activity of chitinases of its host plant (Jashni et al., 2015). Similarly, the serine protease FoSep1 is also essential for the full virulence of *F. oxysporum* against tomato plants (Di Pietro et al., 2001; Jashni et al., 2015). To date, there are no reports about the function of secreted peptidases from *F. oxysporum*.

In the present study, we identified a potential secreted peptidase protein, FoAPY1, which was identified from the secretome of *F. oxysporum* f. sp. *lycopersici*, which causes *Fusarium* wilt disease in tomato plants. FoAPY1 is a member of the peptidase M28 family (aminopeptidase Y, clan MH) and exerts peptidase activity *in vitro*. FoAPY1 can be secreted from the *F. oxysporum* strain and target the nucleus of plant cells. Moreover, deletion of the *FoAPY1* gene significantly decreased the virulence of *F. oxysporum* against tomato plants but had no influence on colony morphology or conidiation. Finally, we also found that FoAPY1 could promote the infection of fungal pathogens in tomato plants. Overall, the results of this study provide insight into the interactions between *F. oxysporum* and host plants and contribute to the understanding of the pathogenic mechanism of this fungus.

## Materials and methods

### Fungal strains and plants growth condition

The *F. oxysporum* f. sp. *lycopersici* strain 4,287 (Ma et al., 2010) and its derivative strains were grown in potato dextrose agar (PDA) at 25°C and all strains were stored at −80°C in 30% glycerine as previously described. The *Botrytis cinerea* strain B05.10 was grown on PDA medium at 25°C. *Escherichia coli* DH5α was cultured in LB medium at 37°C for vector construction, and BL21 (DE3) was used for protein expression. The *Agrobacterium tumefaciens* GV3101 strain was cultured at 28°C and used to agroinfiltration in plants. Tomato AC (Ailsa Craig) and transgenic transformant plants, *Nicotiana benthamiana* were grown under a 16 h light and 8 h dark photoperiod in an artificially controlled growth room at 25°C.

### Plasmids construction

The protein coding sequences of FoAPY1 with or not signal paptides were amplified from the Fo4287 cDNA library using specific primers using the pfu DNA polymerase (TransGen) and then cloned into the pHZ126-Flag vector for secretion assay. For subcellular localization, the cDNA sequences of FoAPY1 without SP was cloned into the Gateway entry vector pQB-V3 and then translated into the destination expression vector cGFP. Using the same method, we construct the vectors to express the recombinant protein FoAPY1-myc or FoAPY1^∆sp^-myc in *N. benthamiana* leaves, respectively.

### Bioinformatics analysis

The signal peptides of all protein were predicted by the SignalP 5.0 server.^1^ The conserved domains of the FoAPY1 proteins were identified using the Pfam database.^2^ And the phylogenetic dendrograms was made using MEGA 5 program.

### Yeast signal sequence trap assay

The yeast secretion system was used for validating the secretion function of the predicted signal peptide. The SP sequences of FoAPY1 and PsAvr1b were cloned into the pSUC2 vector using specific primers for this assay (Lee and Rose, 2012). The constructed vectors and the empty pSUC2 vector were all transformed into the YTK12 yeast strain, respectively. All positive yeast colonies were grown on CMD-W medium (minus Trp). The screened positive colonies were then transferred to YPRAA medium for the invertase secretion assay. Finally, the invertase activity of all the yeast colonies was determined through testing the reduction of TTC to the insoluble, red-colored product triphenylformazan.

### *In vitro* secretion assay

In order to verify the secretion function of the FoAPY1 protein in *F. oxysporum*, the strains of FoAPY1-Flag and FoAPY1^∆sp-Flag^ were cultured in PDB liquid medium for obtaining the conidia. Then, the conidia of two strains and tomato roots were co-cultured, respectively, in 10% YEPD liquid medium at 25°C and 180 rpm for 16 h. The total proteins in culture supernatants were collected after centrifugation using precipitating methods by adding 20% acetone (w/v) and then stored at-80°C for 12 h. Next, the mixed solution was centrifuged at 12,000 *g* at 4°C for 30 min. The total proteins were dissolved in 1× protein loading buffer and then boiling for 10 min. The target proteins were detected by western blotting using anti-flag antibody (1: 10,000, abcam). The anti-actin antibody (1: 5,000, abcam) was used for checking the possibility of the cell lysis during the mycelia growth.

### Deletion and complementation of *FoAPY1*

The *FoAPY1* gene deletion strain (∆FoAPY1) and complemented strain (∆FoAPY1-C) were constructed using PEG-mediated transformation method. For FoAPY1 overexpression strain, the constructed pHZ126-Flag plasmids were transferred into protoplasts of the WT strain. The targeted overexpression strain was detected by western blotting using anti-Flag antibody (1:10,000, abcam). All the derived strains through single-spore isolation to obtain purified strains and then stored at −80°C with 30% glycerine. All primers used in this assay are listed in Supplementary Table S1.

### Plant infection assays

The root-dip method was used to explore the function of FoAPY1 in the virulence of *F. oxysporum* in this assay. Three-week-old tomato seedlings were inoculated with the 10 ml conidial suspension (5.0 × 10^6^ conidia/mL) or with water as a blank control for 20 min each. All tomato seedlings were observed the disease symptoms in 20 days and the disease index was recorded using a previously described method. All infection experiments were repeated three times.

### Agroinfiltration assays

The recombinant plasmids were transformed into *A. tumefaciens* GV3101 through heat shock treatment. This resulting strians were expressed in 4-week-old *N. benthamiana* leaves using a previously described method. For subcellular localization observation, the 48 h after *Agrobacterium*-infiltration the green fluorescence signal was detected using Olympus microscope. The target proteins expressed in the leaves of *N. benthamiana* were confirmed by SDS–PAGE or western blotting.

### Plant manipulation

The cDNA sequence of *FoAPY1* gene was cloned into a construct with the 35S promoter to generate the *FoAPY1* gene transgetic tomato plants. All resulting plasmids were transformed into wild type tomato plants separately using *Agrobacterium*-mediated method as described previously. The targeted transformant plants were confirmed by fluorescence microscopy and western blotting. The T2 transgenic lines were used for further study.

### Infection assay of *Botrytis cinerea* in tomato leaves

The conidia of *B. cinerea* were collected from the PDA plates cultured for 7 days at 25°C and resuspended in the infection buffer (6.7 mm K_2_HPO_4_ and 10 mm glucose). A 5 μl conidial suspension with 5.0 × 10^5^ conidia/mL was dropped onto the surface of all tomato leaves and incubated at 22°C for 60 h, and the disease lesions were measured.

### Prokaryotic expression and purification of FoAYP1

The coding sequence of the full-length or truncated *FoAPY1* gene was cloned into the expression vector pET-28a. The recombinant vector was transformed into *E. coli* BL21 (DE3) for expression. The recombinant proteins were purified using Ni-NAT resin (Beyotime), and the protein concentration was determined using a BCA Protein Assay Kit (Solarbio). The specific primers are listed in Supplementary Table S1.

### Peptide activity assays

The peptidase activity of FoAPY1 was tested by detecting the hydrolysis L-lysine *p*-nitroanilide (Lys-*p*NA) to *p*-nitroanilide with spectrophotometer. The inactivated protein through boiling and buffer was used as negative control in this experiment. The 0.5 mg proteins with 2 mM Lys-*p*NA were incubated at 37°C in buffer containing 50 mM Tris–HCl, pH 8.0. And the changes of absorbance at 405 nm were recorded in 5 min.

### Protein extraction, trypsin digestion and TMT labeling

Total protein of all tomato plants were extracted with cold acetone method (Zhang et al., 2018). The SDS-PAGE was used to examine the protein quality and the BCA Protein Assay Kit was used to determine the protein concentration of all tomato plants. The target proteins were digested using trypsin at 37°C overnight and then the samples were centrifuged and in 500 mM TEAB. The peptides labeled the TMT through TMT 10 Plex™ Isobaric Mass Tag Labeling Kit (Xin et al., 2021).

### Database search, protein identification and annotation analysis

Tandem mass spectra were extracted and transformed into MGF files using Proteome Discoverer 1.2 (Thermo, Pittsburgh, PA, United States) and then further analyzed using the Mascot search engine (Matrix Science, London, United Kingdom; version 2.3.2). The Mascot database was set up for protein identification using the *F. oxysporum* protein information. The Kyoto Encyclopedia of Genes and Genomes (KEGG) database was used to annotate the target protein pathway, and the WoLFPSORT was used to predict the protein subcellular localization. The pathway analysis with a corrected *p* < 0.05 was considered significant.

## Results

### FoAPY1 is a secreted protein from *Fusarium oxysporum*

**Figure 1:**
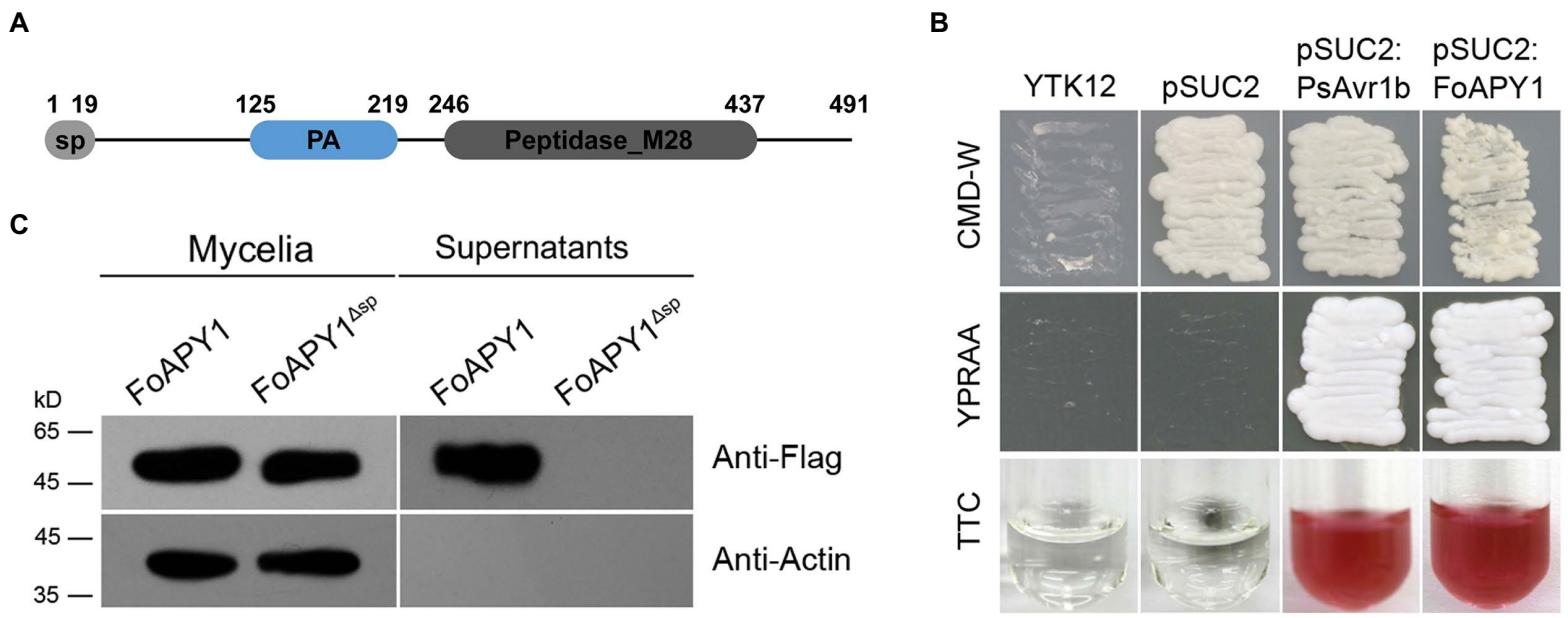
The FoAPY1 protein can be secreted from the *F. oxysporum* strain. **(A)** Diagram of the signal peptide (sp) and domains of the FoAPY1 protein. The sp. was predicted by 5.0 (https://services.healthtech.dtu.dk/service.php?SignalP-5.0) according to the FoAPY1 amino acid sequences. The domains of the FoAPY1 protein were analyzed by the online SMART program (http://smart.embl-heidelberg.de/). **(B)** Secretion functional validation of the predicted sp. of FoAPY1 using a yeast invertase experiment. All the obtained transformed yeast strains were cultured on CMD-W media. YPRAA media were used to verify the yeast invertase-enabled secretion of invertase containing the target sp. sequences in the pSUC2 vector. The YTK12 strain could not grow on CMD-W and YPRAA media due to untransformation of the pSUC2 vector. The change in colour of TTC was used to test the enzymatic activity. The sp. of PsAvr1b from *Phytophthora sojae* was used as a positive control. **(C)** The FoAPY1-Falg and FoAPY1^∆sp^-Falg strains were used for the *in vitro* secretion assay. The conidia of the two strains were cultured in 10% YEPD media and induced with tomato roots. All the proteins in culture supernatants were collected and then analyzed by western blotting in conjunction with anti-Flag (upper panel) and anti-actin (lower panel) antibodies.

In this study, we found a novel secreted protein (FOXG_02417, XP_018235989) from within the *F. oxysporum* secretome (Li et al., 2020), this protein consists of 497 amino acids and is homologous to aminopeptidase Y (APY) of *Saccharomyces cerevisiae*, so we named this protein as FoAPY1. FoAPY1 is believed to contain a predicted 19 amino acid secretion signal peptide at the N-terminus according to SignalP-5.0,^3^ which indicates that FoAPY1 is a predicted secreted protein, in accordance with its existence in the *F. oxysporum* secretome (Figure 1A).

To validate the function of the predicted signal peptide, we used a genetic assay based on the requirement for invertase secretion for yeast growth on raffinose media, of which raffinose is the sole carbon source. The predicted signal peptide region of FoAPY1 and PsAvr1b, which was included as a positive control, were cloned into the yeast vector pSUC2, and then all the resulting constructs were transferred into YTK12 yeast strains. The fusion of both signal peptides to the full-length sequence of SUC2 contributed to the secretion of invertase; the presence of raffinose allowed the transformants to grow on YPRAA media and catalyze the conversion of 2,3,5-triphenyltetrazolium chloride (TTC) to form insoluble red-colored triphenylformazan. In contrast, the two negative controls could not grow on YPRAA media, and the colour of the TTC did not change; it remained colorless under the same conditions (Figure 1B). These results indicated that the predicted signal peptide exerted a secretory function.

To further confirm whether the FoAPY1 protein could be secreted directly from the *F. oxysporum* strains, we generated a FoAPY1 overexpression strain with a Flag tag at the C-terminus of the protein. The FoAPY1-Flag overexpression strain was cultured in 10% YEPD media together with tomato roots to detect the protein in culture supernatants *via* Western blotting using the anti-Flag antibody. The results showed that FoAPY1-Flag proteins were present in the mycelia and culture supernatants. In addition, the Actin protein was detected only in the mycelia, indicating that there was no possibility of cell disruption in the culture media. However, the FoAPY1^Δsp^-Flag protein was not detected in the culture supernatants, although it was successfully expressed in the mycelia of the FoAPY1^Δsp^-Flag overexpression strain. FoAPY1 lost the secretion ability from the *F. oxysporum* strain when the signal peptide was absent, demonstrating that the N-terminal signal peptide is necessary for secretion of this protein (Figure 1C). The results of both yeast genetic assays and secretion assays suggested that, depending on its signal peptide, FoAPY1 is a protein secreted from *F. oxysporum*.

### FoAPY1 possesses peptidase activity and is highly conserved in fungal pathogens

**Figure 2:**
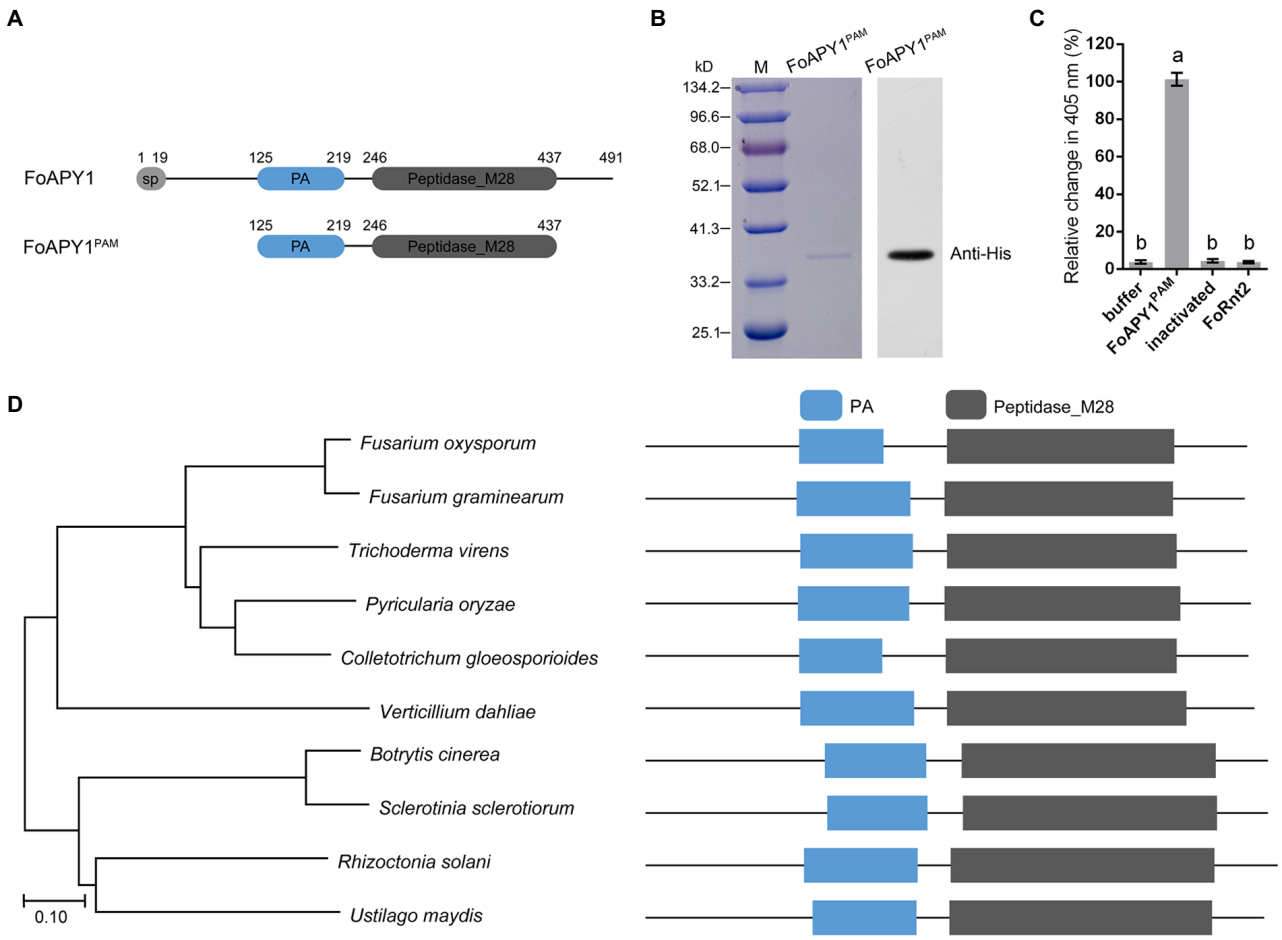
FoAPY1 possesses the peptidase activity. **(A)** Schematic illustration of the FoAPY1 and FoAPY1^PAM^ proteins. **(B)** The protein coding sequences were cloned into the pET-28a vector and then expressed in *E.coli* BL21(DE3). Each protein was incubated with Ni-NTA agarose and then eluted with imidazole. Anti-His was used as the primary antibody in western blotting. **(C)** Quantification of the change in absorbance at 405 nm. Protein buffer and inactivated FoAPY1^PAM^ protein were used as negative controls in this experiment. FoRnt2 was used as another negative control and purified from *E.coli* BL21(DE3) with a His-tag. In the figures, the same letters on the bars mean no significant differences, while the different letters represent significant differences with other groups. **(D)** Phylogenetic analysis of FoAPY1 and its homologous sequences from *B. cinerea* (XP_001553683.1), *Colletotrichum gloeosporioides* (EQB57239.1), *Fusarium graminearum* (XP_011326156.1), *Pyricularia oryzae* (ELQ58420.1), *Sclerotinia sclerotiorum* (XP_001591275.1), *Trichoderma virens* (UKZ76984.1), *Ustilago maydis* (XP_011392725.1), *Verticillium dahliae* (XP_009652286.1) and *Rhizoctonia solani* (CEL55661.1). The all protein domain was predicted by using Pfam database (http://pfam.xfam.org/).

Protein functional prediction and conserved protein domain analysis indicated that FoAPY1 possesses peptidase activity. To test this hypothesis, we expressed the protein in a prokaryotic expression system. Unfortunately, the full-length FoAPY1 protein failed to be expressed. Therefore, we truncated the FoAPY1 protein sequences and then expressed them again in *E. coli* BL21 (DE3; Figure 2A). The results of Coomassie brilliant blue staining and western blotting with anti-His antibody revealedFoAPY1^PAM^ protein (Figure 2B). In accordance with the method described for the aminopeptidase of *Legionella pneumophila* (Zhang N. et al., 2017), L-lysine *p*-nitroanilide (Lys-*p*NA) was used as a specific substrate to detect the peptidase activity in *in vitro* reactions. Lys-*p*NA was liberated to *p*-nitroanilide by peptidase, and the absorbance of the reaction at 405 nm was increased. We recorded the absorbance change after 5 min when Lys-*p*NA was inoculated with the target protein. The absorbance change at 405 nm of the reaction with FoAPY1^PAM^ was very significant compared with that after inoculation with protein buffer and inactivated FoAPY1^PAM^. The results showed that inactivatedFoAPY1^PAM^ and buffer significantly reduced the ability to liberate Lys-*p*NA *in vitro*. Furthermore, a secreted ribonuclease of *F. oxysporum*, FoRnt2, was used as a negative control in this study. Similarly, FoRnt2 did not elicit a significant change in absorbance at 405 nm as inactivated FoAPY1^PAM^ compared with FoAPY1^PAM^ protein under the same conditions (Figure 2C). Taken together, these results suggest that FoAPY1 is a peptidase and has peptidase activity.

To investigate the phylogenetic distribution of FoAPY1 homologs in other pathogens, we performed a protein BLAST search using the FoAPY1 protein sequences in the NCBI database and obtained the identified homologous proteins in different plant pathogenic fungi. Phylogenetic analysis showed that FoAPY1 is conserved in several pathogens, such as the necrotrophic pathogens *B. cinerea* and *S. sclerotiorum*, and the homologous proteins contain a PA and peptidase domain (Figure 2D). Taken together, our results show that FoAPY1 exerts peptidase activity and is conserved in different pathogens.

### FoAPY1 is not involved in mycelial growth or conidiation

To investigate the biological function of the *FoAPY1* gene, we used the split-marker method to generate a gene replacement construct containing an *hph* gene and transformed the construct into protoplasts of the wild-type (WT) strain Fo4287. Two deletion mutants (∆FoAPY1-1 and ∆FoAPY1-2) were identified from the candidate hygromycin-resistant transformants *via* PCR with the primers listed in Supplementary Table S1. To further confirm that the change in ∆FoAPY1 strains occurred because of the *FoAPY1* gene deletion, we transformed protoplasts of the ∆FoAPY1 strain again by introducing the FoAPY1-GFP construct with the self-promoter and terminator region sequences to obtain a complementation strain (∆FoAPY1-C; Supplementary Figure S1).

**Figure 3:**
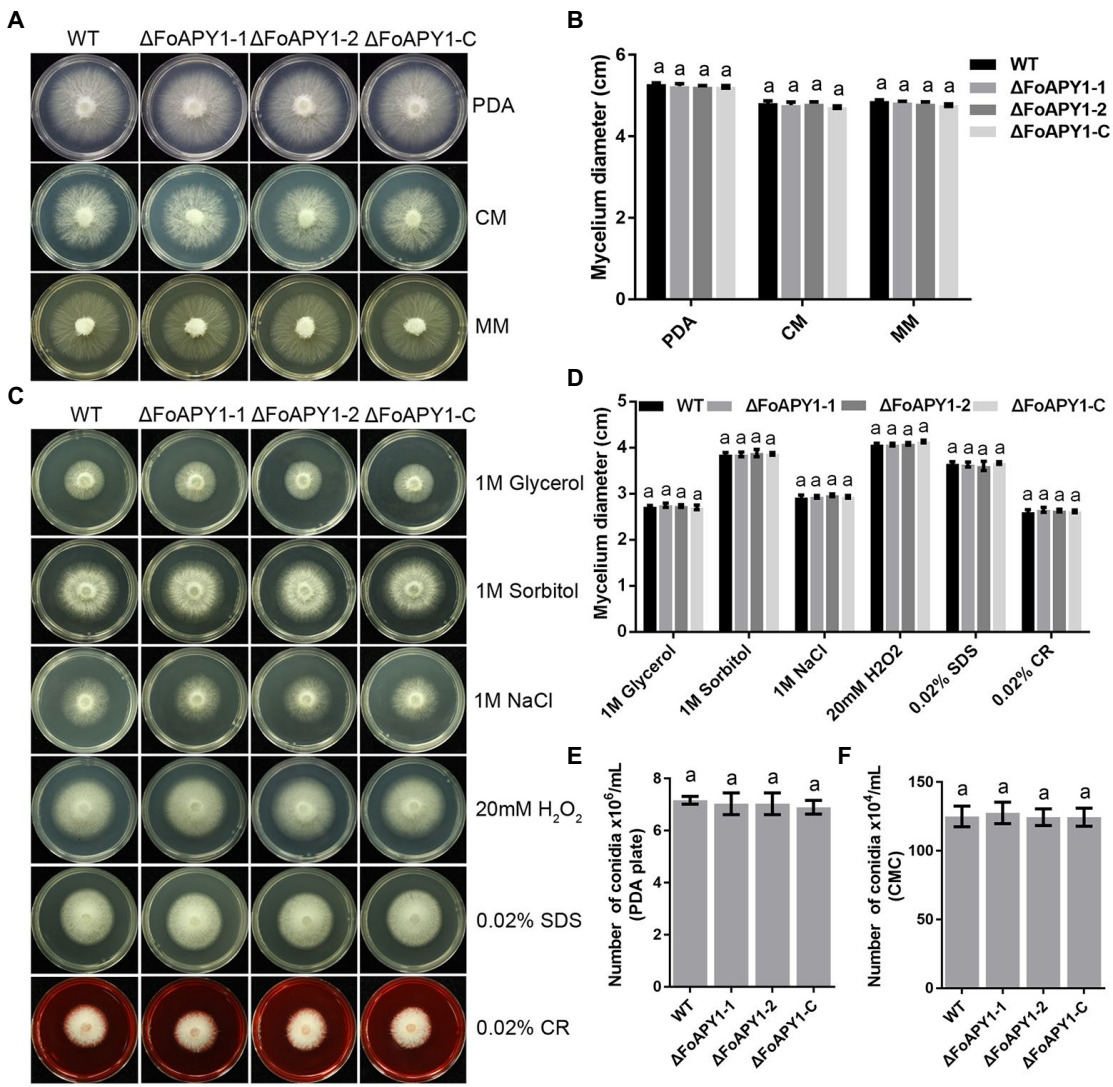
The FoAPY1 is not essential for mycelial growth or conidiation of *F. oxysporum*. **(A)** Mycelial growth of WT, two ∆FoAPY1 mutant and ∆FoAPY1-C complementation strains grown on PDA media, CM and MM at 25°C for 3 days in the dark. **(B)** Quantification of colony diameters for the WT strain, ∆FoAPY1 mutant, and ∆FoAPY1-C complementation strain cultured on PDA media, CM and MM after 3 days. **(C)** Mycelial growth of all strains on PDA media amended with 1 M glycerol, 1 M sorbitol, 1 M NaCl, 20 mM H_2_O_2_, 0.02% SDS, and 0.02% CR. All the strains were cultured at 25°C in the dark, and images were taken after incubation for 3 days. **(D)** Quantification of colony diameters for all targeted strains growing from the mycelial plug on PDA media with the different stress-mimicking agents. **(E)** The number of conidia of the WT, ΔFoAPY1 mutant and ∆FoAPY1-C complementation strains was measured after 6 days of incubation on PDA media at 25°C. The conidia were harvested using ddH_2_O and then filtered through lens paper. **(F)** Conidia number of all the strains cultured in CMC liquid media at 180 rpm at 25°C for 2 days. All the experiments were performed three times in this study. The collected data were analyzed by one-way ANOVA for statistical tests. In the figures, the same letters on the bars mean no significant differences.

We then compared the phenotypes of the ∆FoAPY1 strains with those of the WT and ∆FoAPY1-C strains to determine the potential function of FoAPY1. No difference in colony morphology were found among the ∆FoAPY1, WT and ∆FoAPY1-C strains on potato dextrose agar (PDA) media, complete media (CM) and minimal media (MM; Figure 3A). In addition, the growth rate of the ∆FoAPY1 strains was similar to that of the WT and ∆FoAPY1-C strains on media under the same conditions (Figure 3B). To test whether deletion of FoAPY1 could mediate *F. oxysporum* adaptation to various stresses, we exposed the ∆FoAPY1, WT and ∆FoAPY1-C strains to PDA media containing the osmotic stress agents glycerol, sorbitol and NaCl; the oxidizing stress agent H_2_O_2_; and the cell wall-damaging agents sodium dodecyl sulfate (SDS) and Congo red (CR).The mycelial growth rate of those strains under each stress did not show any difference, indicating that FoAPY1 is not involved in osmotic and oxidative stress and does not affect cell wall integrity (Figures 3C,D). To evaluate the role of FoAPY1 in conidiation, we also inoculated mycelial plugs of all strains in carboxymethyl cellulose (CMC) liquid media for 2 days. Our quantitative data showed that loss of the *FoAPY1* gene did not affect the conidiation of the ∆FoAPY1 strain compared with the WT and ∆FoAPY1-C strains (Figure 3F). Further observations of conidiation in PDA media demonstrated that the number of conidia of all the tested strains did not show any difference, suggesting that FoAPY1 is not required for conidiation of *F. oxysporum* (Figure 3E). Taken together, our results revealed that FoAPY1 did not play a part in mycelial growth or adaptation to various stresses and was not required for conidiation.

### FoAPY1 is essential for the virulence of *Fusarium oxysporum*

**Figure 4:**
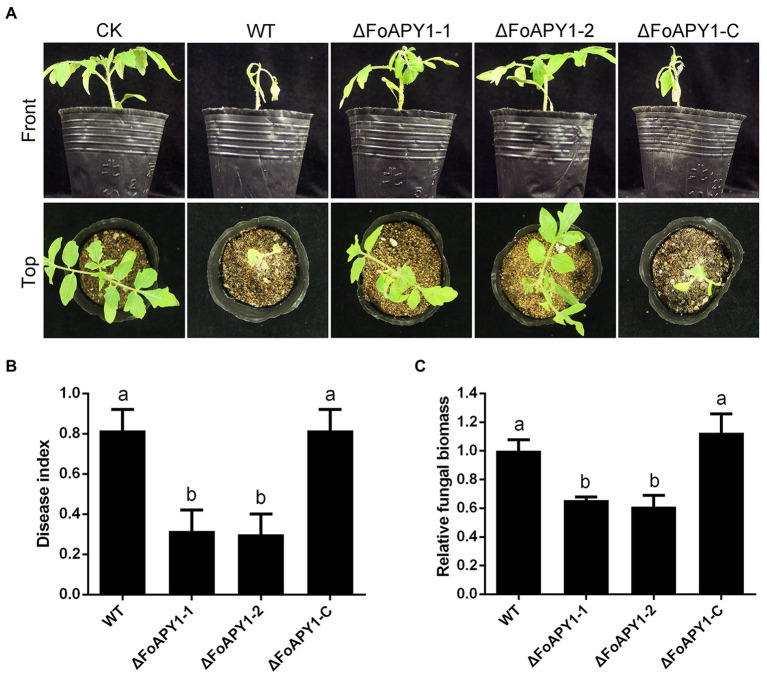
FoAPY1 is required for full virulence of *F. oxysporum*. **(A)** Pathogenicity assays on tomato seedlings inoculated with the conidial suspension of WT, two ∆FoAPY1 mutant and ∆FoAPY1-C complementation strains and imaged 20 days after inoculation. CK represents the plants treated with water as a blank control. The infection experiments were repeated three times. **(B)** Quantification of the disease indexes of the plants inoculated with all indicated strains. The disease index was calculated using the formula: disease index = Σ (number of plant leaves × grade value)/(total number of leaves × maximum grade value). **(C)** qRT–PCR analysis of fungal biomass in all plants infected by all *F. oxysporum* strains in this study. The relative fungal biomasses of the WT strain were set to values of 1. The constitutively expressed gene *Sl18S* was used as an internal reference. The experiment was performed for three replicates, and the average values were calculated. The different letters above the bars means mean significant differences at *p* < 0.05.

To investigate the possible function of FoAPY1 in *F. oxysporum* virulence, we observed changes in symptoms of tomato seedlings during inoculation. Three-week-old seedlings were inoculated with conidia of the WT, ∆FoAPY1 and ∆FoAPY1-C strains at 25°C for 20 days. Obvious disease symptoms such as stunted plant growth and leaf yellowing occurred for tomato seedlings inoculated with the WT and ∆FoAPY1-C strains, all of which showed higher infection ability, whereas significantly reduced disease symptoms were detected in tomato seedlings inoculated with the two ∆FoAPY1 strains (Figure 4A). The disease symptom severity of tomato seedlings inoculated with the WT and ∆FoAPY1-C strains increased rapidly with increasing days of inoculation, while the disease symptom severity of tomato seedlings inoculated with the ∆FoAPY1 strains was significantly delayed compared with that of seedlings inoculated with the WT and ∆FoAPY1-C strains under the same inoculation conditions (Figure 4B). To further determine the function of FoAPY1 in virulence, we quantified the fungal biomass of *F. oxysporum* in roots infected by all the tested strains. The results showed that, compared with the plants inoculated with ∆FoAPY1 strains, the plants inoculated with WT and ∆FoAPY1-C strains led to a significant increase in fungal biomass *in planta* (Figure 4C). These results indicated that FoAPY1 is required for the virulence of *F. oxysporum.*

### FoAPY1 is localized in the nucleus in plant cells

**Figure 5:**
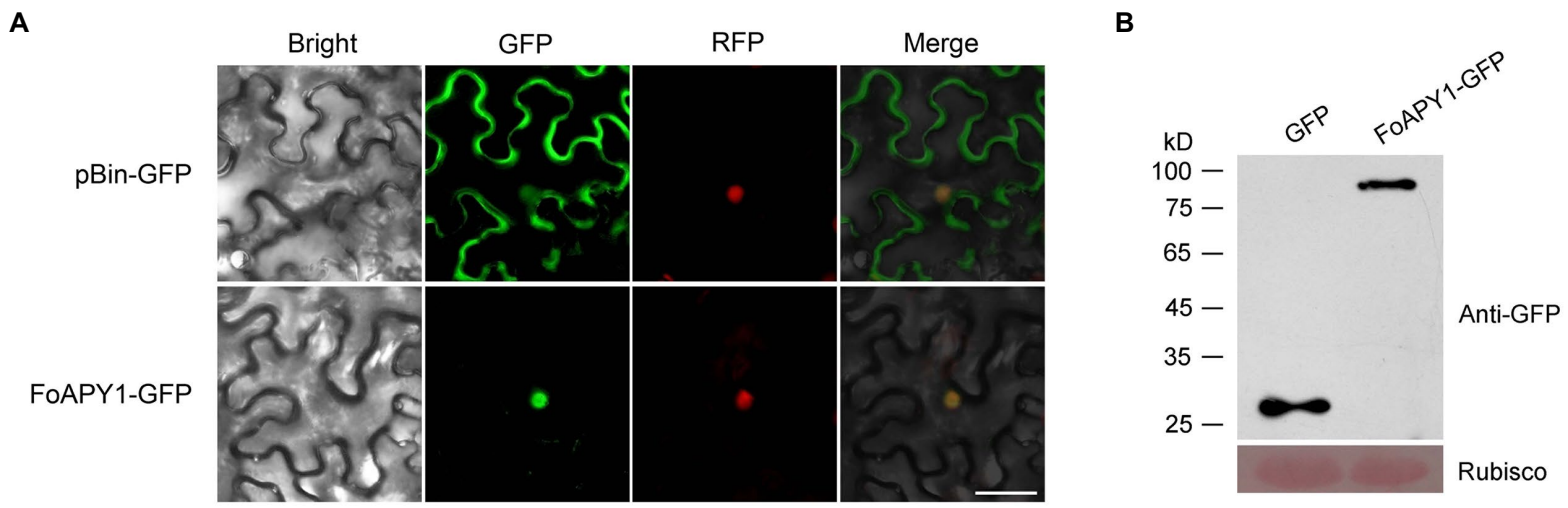
Subcellular localization of FoAPY1 in *N. benthamiana*. (A) Fluorescence microscopy analysis of *N. benthamiana* leaves subjected to *Agrobacterium*-mediated *N. benthamiana* transient expression of FoAPY1-GFP fusion protein (without signal peptide). The GFP tag alone was expressed in *N. benthamiana* leaves as a control. H2B-RFP (red fluorescent protein) was used for nucleus localization. All the images were taken at 48 h post-agroinfiltration. Bar = 20 μm. **(B)** The proteins of *N. benthamiana* leaves expressed GFP or FoAPY1-GFP were analyzed by western blotting with Anti-GFP antibody. Equal loading amounts of protein were confirmed by staining Rubisco with Ponceau S.

To explore the localization and biological function of FoAPY1, we transiently expressed FoAPY1-GFP fusion proteins, in which green fluorescent protein (GFP) was added to the target protein C-terminal end, in *N. benthamiana* leaves to determine subcellular localization using *A. tumefaciens* infiltration and observed the GFP signal at 48 h. FoAPY1-GFP was detected in the plant nucleus, and nuclear localization was confirmed by H2B, which was tagged with RFP and localized in the nucleus of *N. benthamiana* cells. The fluorescent signal distribution showed that green and red fluorescent protein signals overlapped in the nucleus (Figure 5A). The mature protein was determined by western blotting analysis using an anti-GFP antibody (Figure 5B). These results indicated that the secreted FoAPY1 protein probably localized to the nucleus of plant cells.

### Proteomic identification and analysis of *FoAPY1* transgenic tomato plants

**Figure 6:**
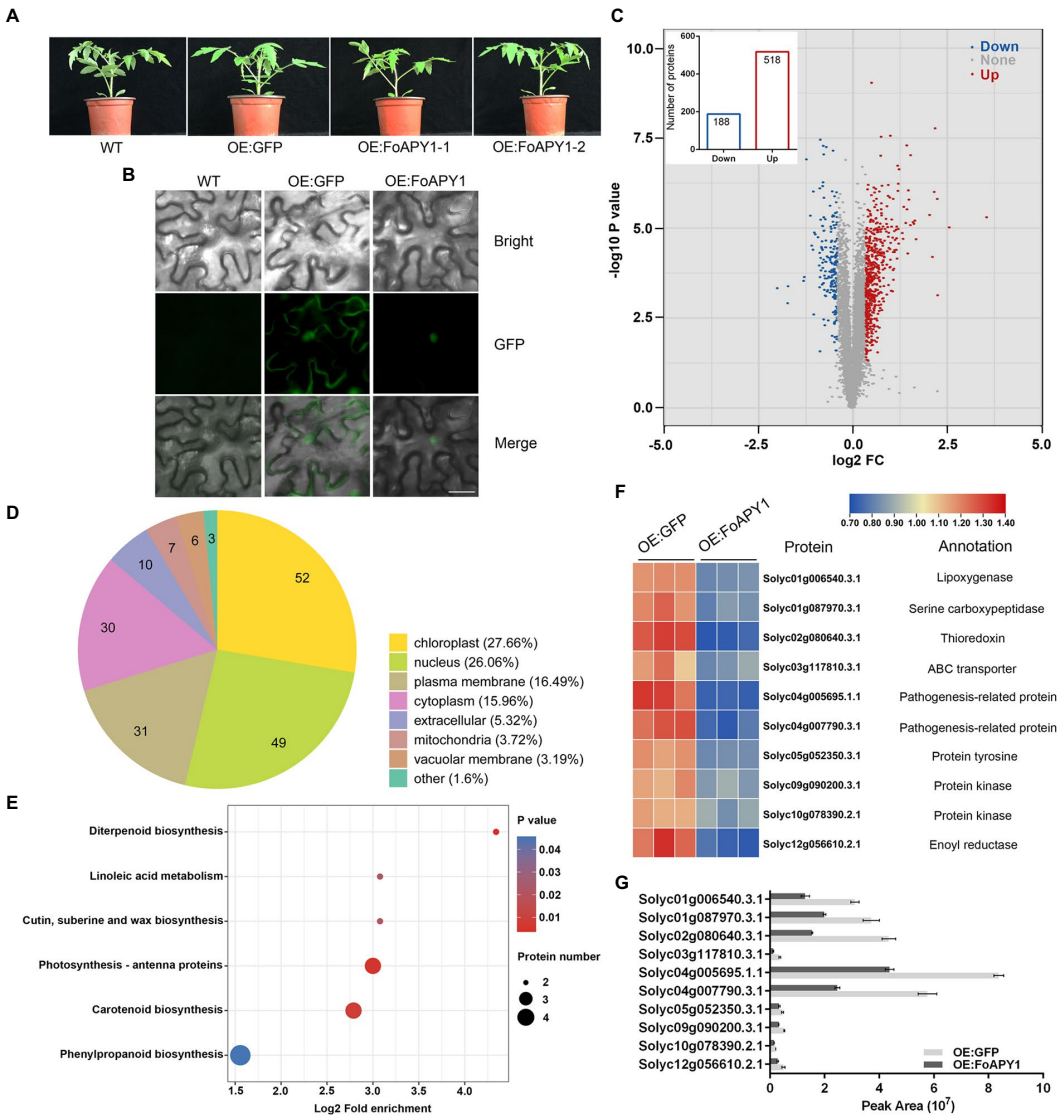
Proteomic analysis of *FoAPY1* gene transgenic tomato plants. **(A)** Morphology of WT, OE:GFP and OE:FoAPY1 tomato plants. All the plants were grown at 25°C for 4 weeks in an artificially controlled growth chamber, and images were collected. **(B)** Subcellular localization of FoAPY1 protein fused with a GFP tag in tomato (*Solanum lycopersicum*). GFP alone was used as a negative control in this experiment. Bars = 20 μm. **(C)** Volcano plot of DAPs identified in the OE:GFP and OE:FoAPY1-GFP plant groups. The blue dots represent the downregulated proteins, the red dots represent the upregulated proteins, and the gray dots represent the proteins with no significant change in the two groups. The number of DAPs is shown in the histogram. **(D)** Subcellular localization classification of the downregulated proteins was predicted. **(E)** KEGG pathway enrichment analysis of downregulated proteins. All the proteins were enriched in six pathways. The x-axis represents the enrichment factor; the y-axis represents the main KEGG pathways. The point size indicates the number of target genes. **(F)** Expression level analysis of selected DAPs in OE:GFP and OE:FoAPY1 tomato plants. The proteins are displayed in different colors. Protein abundance levels are shown in a colour gradient from low to high as a legend. Each heatmap from left to right shows OE:GFP and OE:FoAPY1. The annotation of each protein is shown on the right of the heatmap. **(G)** Ten selected proteins in the proteome of different groups measured by PRM. The peak area represents the protein abundance in each tomato plant sample.

To better understand the function of invasive peptidase in host plants from pathogens, *FoAPY1* transgenic tomato plants (OE:FoAPY1) and control plants (OE:GFP) were obtained. Compared with the WT tomato plants, the OE:FoAPY1 and OE:GFP tomato plants exhibited no morphological differences (Figure 6A). Western blotting suggested that the FoAPY1 protein with a GFP tag was successfully expressed in tomato plants (Supplementary Figure S2). FoAPY1 was mainly localized in the nucleus of tomato cells according to microscopy observations of green fluorescent signals, and this result is consistent with its localization in *N. benthamiana* cells (Figure 6B). The proteins that accumulated more than 1.3 or less than 0.77 were termed differentially abundant proteins (DAPs) within the protein dataset. These proteins presented a significant increase or decrease in accumulation (in terms of fold-change; *p* < 0.05). In this study, a total of 706 DAPs in the above two types of tomato plants were obtained, including 518 upregulated proteins and 188 downregulated proteins (Figure 6C). The downregulated proteins were widely distributed in various organelles, indicating that FoAPY1 may play diverse roles in different cell organelles (Figure 6D; Supplementary Table S2). In addition, using the Kyoto Encyclopedia of Genes and Genomes (KEGG) database, we found that downregulated proteins in the DAPs were enriched in six pathways, which are mainly involved in biosynthesis and metabolism (Figure 6E; Supplementary Table S3). Among the downregulated proteins, some pathogenesis-related (PR) proteins and plant protein kinase are related to the host defense response to pathogens. Ten downregulated proteins were randomly selected in enriched pathways for the analysis of protein abundance. The protein abundance of ten candidate proteins all decreased in the OE:FoAPY1 tomato plants compared with the control plants(Figure 6F). The ten selected proteins in the proteomic profile were measured again by the parallel reaction monitoring (PRM) method. The results showed that all the proteins showed decreasing trends according to the PRM analysis, and the findings of which were essentially consistent with the proteomic profiling data (Figure 6G), suggesting the relative rationality and accuracy of proteomics between the two plant groups. Proteomic analysis of OE:FoAPY1 tomato plants indicated that FoAPY1 plays a role in tomato by altering the abundance of proteins in different pathways and diverse organelles.

### FoAPY1 enhances tomato susceptibility to fungal pathogens

**Figure 7:**
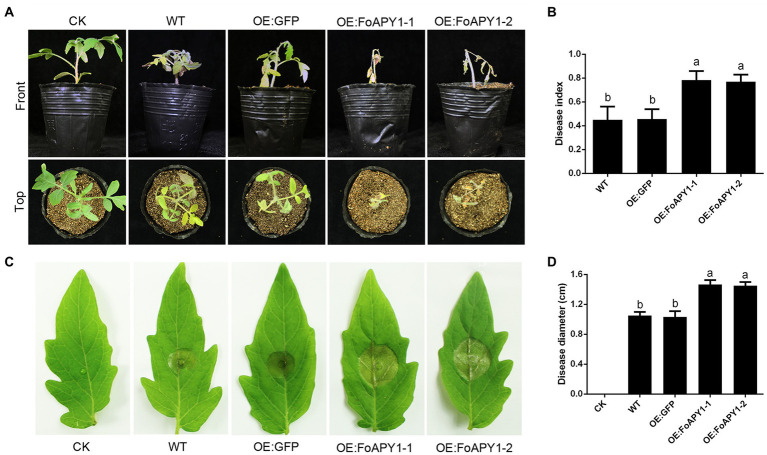
FoAPY1 promotes the infection of fungal pathogens in tomato plants. **(A)** Phenotypes of WT, OE:GFP and OE:FoAPY1tomato seedlings inoculated with conidia of *F. oxysporum* at 5 × 10^6^ conidia/mL. The images were taken at 16 days post-inoculation. CK represents the tomato seedlings treated with water. **(B)** The record of disease index of all the tomato seedlings to represent the disease severity. The disease severity was defined from 1 to 5 according to the disease degree, and 0 represents no disease symptoms. **(C)** The disease phenotypes of tomato leaves inoculated with conidia of *B. cinerea*. Five microlitres of conidial suspension at 5.0 × 10^5^ conidia/mL was dropped onto the surface of all tomato leaves and incubated at 22°C for 60 h. The disease lesions on different types of tomato leaves were recorded, and the negative control was performed on WT leaves with infection buffer (6.7 mm K_2_HPO_4_ and 10 mM glucose). **(D)** Quantification of lesion diameter on tomato leaves inoculated with *B. cinerea*. Three repeated experiments were performed, and ten leaves of different types of tomato were used in each experiment. All the data were calculated by one-way ANOVA for statistical tests. The same letters marked on the bars mean no significant differences, and the different letters indicate significant differences in values at *p* < 0.05 in the figure.

To further explore FoAPY1 function in plants and examine whether FoAPY1 could modulate host plant resistance to *F. oxysporum*, WT tomato plants and *FoAPY1* transgenic plants (OE:FoAPY1) were used to identify susceptibility to *F. oxysporum*. All of the tomato seedlings, including the control plants (OE:GFP), were inoculated with conidia of *F. oxysporum* using the root-dip method. Sixteen days post-inoculation (dpi), all the plants inoculated with *F. oxysporum* showed different disease symptoms and were imaged (Figure 7A). The *FoAPY1* transgenic seedlings (OE:FoAPY1) showed more obvious symptoms than WT and OE:GFP tomato seedlings under the same conditions (Figure 7B). In addition, *B. cinerea*, which is a pathogen of tomato, was also used to infect tomato leaves in this study. Similarly, the lesion sizes of leaves in which the FoAPY1 protein was overexpressed were significantly larger than those of the control plant leaves (Figures 7C,D). These results indicated that FoAPY1 enhances host plant susceptibility to pathogens and that FoAPY1 expressed in the host plant significantly promotes fungal pathogen invasion.

## Discussion

*F. oxysporum* is a fungal pathogen that causes root rot and wilting disease in more than 150 host plants. Similar to the fungal pathogen *Verticillium dahlia* (de Sain and Rep, 2015; Zhang Y. et al., 2021) and the pathogenic oomycete *Phytophthora sojae* (Ai et al., 2021; Wang et al., 2021) which can secrete many proteins during infection, *F. oxysporum* can also secrete some proteins to promote effective pathogen infection of host plants, such as secreted xylem (Six) proteins and other characterized proteins, which have been shown to be important for pathogenicity(Houterman et al., 2007; Gawehns et al., 2015). Six 1 proteins are essential for the virulence of *F. oxysporum* and are targets for the resistance gene *I-3* of tomato (Widinugraheni et al., 2018). Similarly, the effector protein Six 6 is also required for virulence and can suppress host cell death (Gawehns et al., 2014). Moreover, the secreted polygalacturonases (PGs) protein PG1 and PG6 are required for the virulence of *F. oxysporum* to host plants depending on their activity (de Sain and Rep, 2015; Bravo Ruiz et al., 2016). In addition, a small group of uncharacterized proteins are secreted to plant cells from *F. oxysporum*. Fosp9, a small protein containing unknown motifs and domains, is a novel secreted protein that is important for the full virulence of *F. oxysporum* (Guo et al., 2022).

In this study, we focused on the secreted protein FoAPY1 from the *F. oxysporum* secretome; this protein has a peptidase domain and belongs to the peptidase M28 family. Using different methods, we proved that, depending on its signal peptide at the N-terminus, FoAPY1 is a secreted protein. Deletion of the *FoAPY1* gene significantly decreases the virulence of this pathogen to tomato plants, indicating that FoAPY1 is a secreted protein and is involved in the virulence of *F. oxysporum*.

The secreted protein aminopeptidases AO-LapA of *A. oryzae* (Baltulionis et al., 2021) and leucine aminopeptidase rLap1 from *A. sojae* (Huang et al., 2015) are the members of the peptidase M28 family and are widely used in food industry to promote proteolysis (Matsui et al., 2006). In addition, the aminopeptidase Y (APY) of *S. cerevisiae* is essential for the vacuolar proteolytic processing system and plays important roles in biological processes (Ai et al., 2021). However, the biological function of the secreted peptidase of *F. oxysporum* is poorly understood. To further explore the potential functions of secreted peptidase FoAPY1, a FoAPY1^PAM^-His (containing the PA and peptidase domains) fusion construct was expressed in *E. coli*, and the specific activity substrate Lys-*p*NA was used for enzymatic activity examination. As the aminopeptidase LapB from *L. pneumophila* has possesses peptidase activity through catalytic Lys-*p*NA substrate (Zhang N. et al., 2017), FoAPY1^PAM^ could also liberate Lys-*p*NA *in vitro*. These results indicated that FoAPY1 exerts a peptidase function similar to that of the other member of the peptidase M28 family.

When pathogens infect and secrete effectors to host cells, the defense responses of plants, such as reactive oxygen species (ROS) bursts, resistance gene expression and cell death, are activated to inhibit pathogen growth and development. SsCP1 is recognized by the PR1 protein of the host plant to trigger defense responses when *S. sclerotiorum* infects the host plant (Yang et al., 2018). Expression of VdSCP7, a *Verticillium*-specific protein, in plants has been shown to induce ROS accumulation, resulting in activation of plant host plant defense responses (Zhang Y. et al., 2017). The secreted ribonuclease Zt6 from *Zymoseptoria tritici* can induces the cell death of *N. benthamiana* (Kettles et al., 2018). Moreover, SsSSVP1can cause significant cell death when it is expressed in plants (Lyu et al., 2016). In our study, transient expression of FoAPY1 protein in *N. benthamiana* did not induce cell death (Supplementary Figure S3), similar to the findings of effector CsSp1 from *Bipolaris sorokiniana*, which does not have the ability to induce cell death. The results indicated that the FoAPY1 protein is not similar to other effectors in terms of its cytotoxic effects in the plant or may not be recognized in the host cell.

Expression of the *Phytophthora infestans* RXLR effector PITG20303 in plants promotes this pathogen infection by targeting the MKK1 protein to inhibit the immune response of the host (Du et al., 2021). VdSCP7 of *V. dahliae* enhanced susceptibility to *Phytophthora capsicum* mainly by modifying the host plant immune response (Zhang Y. et al., 2017). Similarly, by inhibiting GhPR5 activity to overcome host defense, the Alt a 1-like protein PevD1 of *V. dahliae* could also promote fungal infection (Zhang et al., 2019b). In this study, using the *Agrobacterium*-mediated method, we overexpressed the FoAPY1 protein in tomato plants and then inoculated the plants with the conidia of *F. oxysporum* and *B. cinerea*. The results showed that FoAPY1 significantly enhances susceptibility to fungal pathogens and promotes invasion, suggesting that FoAPY1 may suppress the defense response of host plants during *F. oxysporum* infection.

To explore the potential functional mechanism of the secreted protein FoAPY1 and the host plant, proteomic analysis was performed on OE:GFP and OE:FoAPY1 tomato plants. Since FoAPY1 exerts peptidase activity and may alter the abundance of plant proteins, we mainly focused on the downregulated proteins. The abundances of some Pathogenesis-related (PR) proteins were decreased in the OE:FoAPY1 plant compared the OE:GFP plant. PR proteins play important roles in the plant immune system during the host plant recognition of pathogens (Zhou et al., 2021). PR5 of tobacco has antifungal activity, and overexpression of PR5 in tobacco could enhance resistance to *P. infestans* (Tjamos et al., 2005; Zhang et al., 2019a). Two PR proteins, MdPR10-1 and MdPR10-2, of apple plants promote resistance to *Alternaria alternata* (Zhang Q. et al., 2021). In addition, the genes of the PR1, PR2, PR4, and PR5 families of garlic are essential for defense against *Fusarium* infection (Anisimova et al., 2021). Some PR proteins were downregulated in FoAPY1-overexpressing tomato plants compared to control plants. Furthermore, we also found that the protein abundances of several protein kinases were decreased. Plant protein kinase plays crucial roles in plant immunity to defend against pathogen infection (Akira et al., 2006; Tena et al., 2011; Shen and Hanley-Bowdoin, 2021). In addition, FoAPY1 is required for the virulence and targets the cell nucleus of plants, and the abundance of some proteins in the nucleus were decreased when FoAPY1 was expressed in tomato plants demonstrating that FoAPY1 may change the abundances of proteins in nucleus and then suppress the host plant defense system for contribute to the virulence of *F. oxysporum* to plants. Furthermore, FoAPY1 could promote fungal infection in tomato plants and increase the susceptibility to *F. oxysporum* and *B. cinerea* by degrading several proteins, and based on the peptidase activity, may be involved in plant resistance to pathogens during infection.

## Conclusion

The results of this study demonstrated that FoAPY1 is required for the full virulence of *F. oxysporum* against host plants and exhibits peptidase activity. The overexpression of FoAPY1 in tomato plants promoting the infection of *F. oxysporum* and *B. cinerea* may depend on the enzymatic activity of the FoAPY1 itself. The results of this study help explain the biological function of peptidases in plant pathogenic fungi and are helpful for understanding the pathogenic mechanism of *F. oxysporum*.

## Data availability statement

The original contributions presented in the study are included in the article/Supplementary material, further inquiries can be directed to the corresponding author.

## Author contributions

WL designed research. HQ and LW performed research. LW and LS contributed new reagents or analytic tools. HQ, LS, BW, and WL analyzed data. HQ and WL wrote the paper. All authors contributed to the article and approved the submitted version.

## Funding

This research was funded by the National Natural Science Foundation of China (31972213), the Natural Science Foundation of Shandong Province (ZR2020KC003), the Key Research and Development Program of Shandong Province (2019YQ017), Shandong Province “Double-Hundred Talent Plan” (WST2018008), and Taishan Scholar Construction Foundation of Shandong Province (tshw20130963).

## Conflict of interest

The authors declare that the research was conducted in the absence of any commercial or financial relationships that could be construed as a potential conflict of interest.

## Publisher’s note

All claims expressed in this article are solely those of the authors and do not necessarily represent those of their affiliated organizations, or those of the publisher, the editors and the reviewers. Any product that may be evaluated in this article, or claim that may be made by its manufacturer, is not guaranteed or endorsed by the publisher.

## Footnotes

^1^ http://www.cbs.dtu.dk/services/SignalP/

^2^ http://pfam.xfam.org/

^3^ http://www.cbs.dtu.dk/services/SignalP/
