# Supplementary figures and images for: The secreted FoAPY1 peptidase promotes Fusarium oxysporum invasion
Source: Front Microbiol. 2022 Oct 19;13:1040302. doi: 10.3389/fmicb.2022.1040302 (PMC9626516; doi:10.3389/fmicb.2022.1040302)

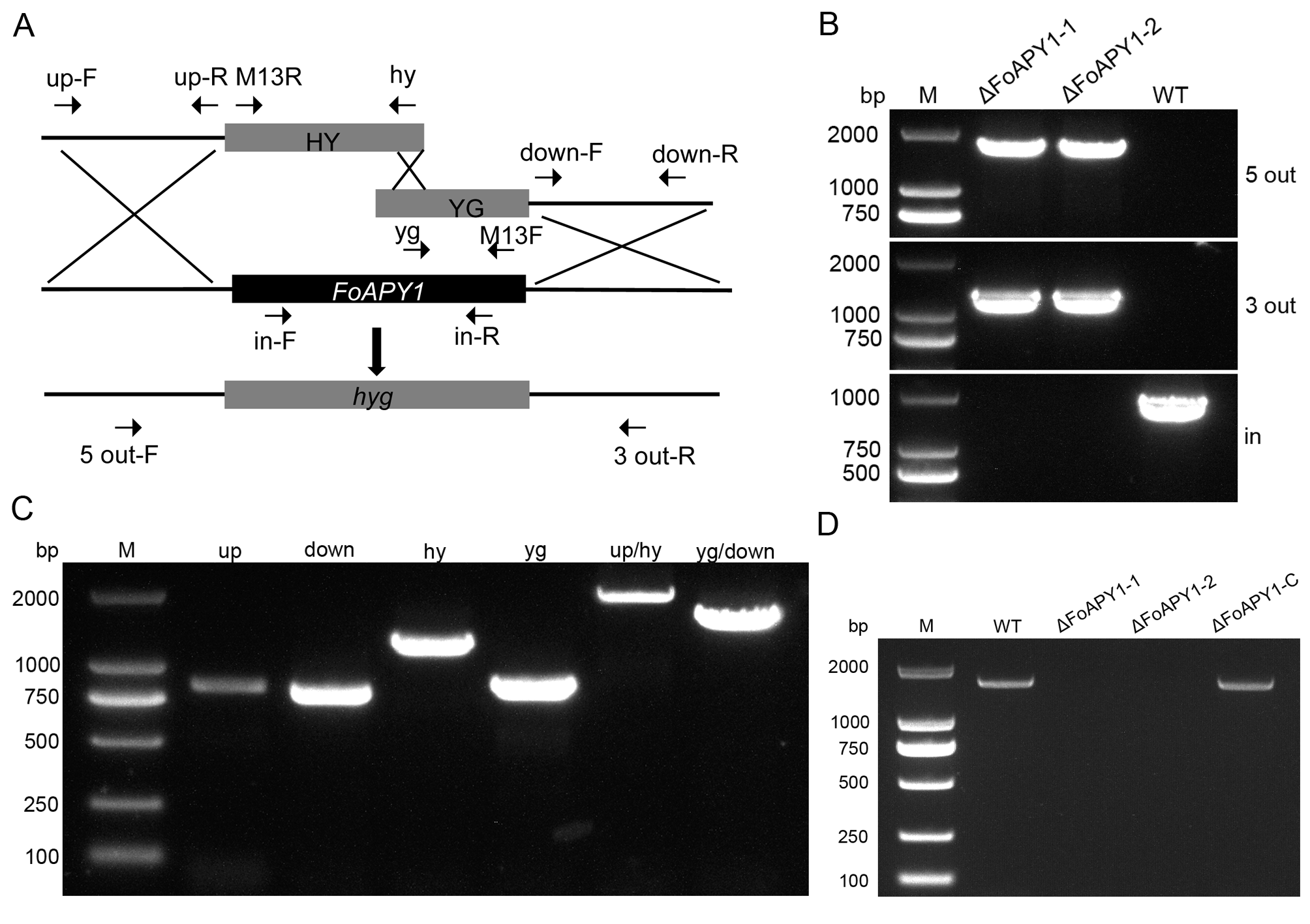

Supplement: Supplementary file 1 [file Image_1.TIF]

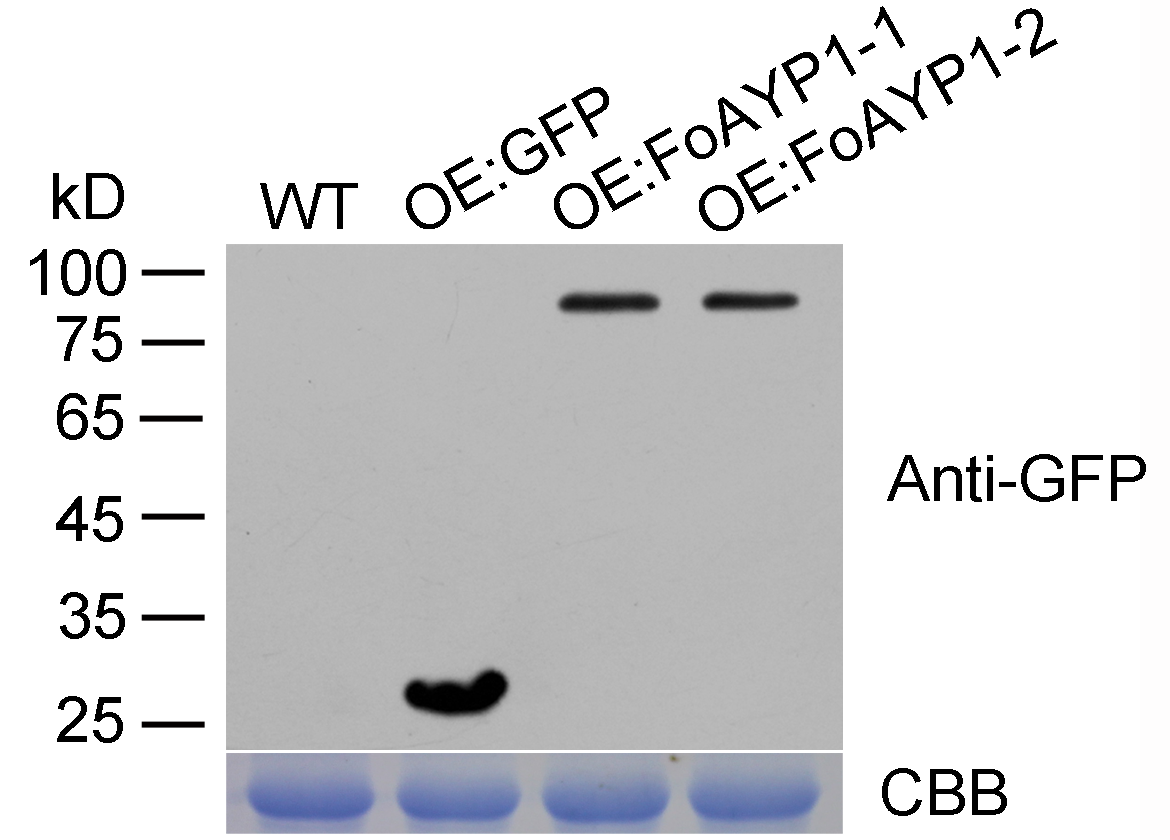

Supplement: Supplementary file 2 [file Image_2.TIF]

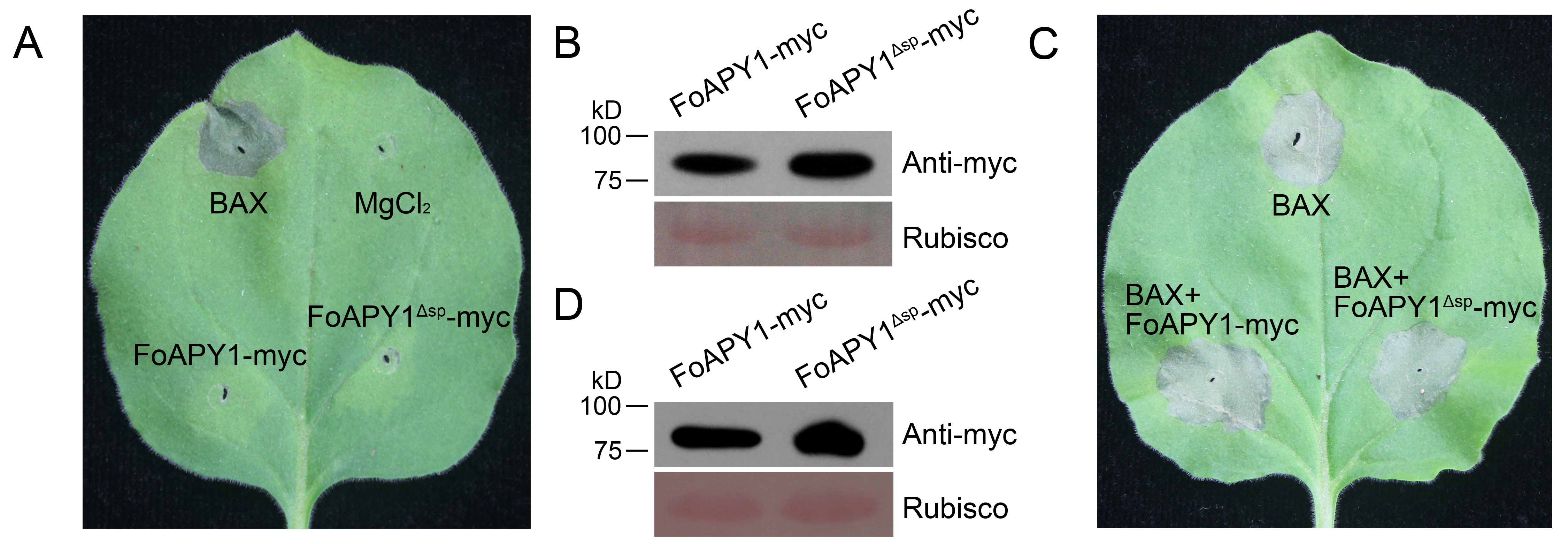

Supplement: Supplementary file 3 [file Image_3.TIF]
