# Supplementary material for: The secreted FoAPY1 peptidase promotes Fusarium oxysporum invasion
Source: Front Microbiol. 2022 Oct 19;13:1040302. doi: 10.3389/fmicb.2022.1040302 (PMC9626516; doi:10.3389/fmicb.2022.1040302)
Supplement: Supplementary file 4 [file Table_1.DOCX]

**Primers used in this study**

| **Primer** | **Sequence (5’-3’)** | **Application** |
| --- | --- | --- |
| FoAPY1-up-F | AACCGAAAGC CGTTGGGTTA CC | Up flank of *FoAPY1* for deletion |
| FoAPY1-up-R | TCCTGTGTGAAATTGTTATCCGCTGAGTAACAATG CCTCTAGAAG ACG |  |
| FoAPY1-down-F | :GTCGTGACTGGGAAAACCCTGGCG AAAGCGTTGG CGGAGAGGAA TG | Down flank of *FoAPY1* for deletion |
| FoAPY1-down-R | CCGCCAAGAGG TGCAGGTTAT |  |
| M13F | CGCCAGGGTTTTCCCAGTCACGAC | Split marker |
| M13R | AGCGGATAACAATTTCACACAGG | Split marker |
| HY | GGATGCCTCCGCTCGAAGTA | Upflank of*hph* genewith M13R |
| YG | CGTTGCAAGACCTGCCTGAA | Downflank of*hph* genewith M13F |
| FoAPY1-5-out | GA CCTGCCATTT TCGCATCGTC | Confirming PCR of FoAPY1 deletion mutants |
| FoAPY1-3-out | CAGTCAAGGCG TCAGATGGGA |  |
| GC1 | ACTTCTCGACAGACGTCGC | Confirming PCR of*hph*gene |
| GC2 | TGGCTGTGTAGAAGTACTCG |  |
| FoAPY1-in-F | ATGAAGTTAC TTAGTGCTTT GCTCTGC | Confirming PCR of *FoAPY1*gene |
| FoAPY1-in-R | GACAACAACC TTTGTCTTAT GGTTAC |  |
| FoAPY1-com-F | GATATCGAATTCCTGCAGGACATCTTCTCGGGGAGTGT GAT | Construct the *FoAPY1* complement strain |
| FoAPY1-com-R | CCACCGCGGTGGCGGCCGC GCTGCCGAA GACCGAAGCA G |  |
| FoAPY1^Δsp^-Flag-F | TTTCGTAGGAACCCAATCTTCAAAATGAAGTTAC TTAGTGCTTT GCTCTGC | Construct the *FoAPY1* overexpression strain |
| FoAPY1-Flag-F | TTTCGTAGGAACCCAATCTTCAAA ATGACCAAGAAGC TGACTCCCAG TC |  |
| FoAPY1-Flag-R | CTTTATAATCACCGTCATGGTCTTTGTAGTCGACAACAACC TTTGTCTTAT GGTTAC |  |
| FoAPY1-psuc2-F | CACCACCCCGGTGAACAGCTCCTCGCCCTTGCTCACGACAACAACC TTTGTCTTAT GGTTAC | Construct pSUC2 vector for YTK12 strain |
| FoAPY1-psuc2-R | AATTCATGAAGTTAC TTAGTGCTTT GCTCTGCGGA GCTCTCCTCT TCTCTGAGGT GTCGGCT C |  |
| pQB-FoAPY1-F | ATGAAGTTAC TTAGTGCTTT GCTCTGC | For expression the FoAPY1-GFP or myc in plant |
| pQB-FoAPY1^Δsp^-F | ATGACCAAGAAGC TGACTCCCAG TC | For expression the FoAPY1^Δsp^-GFP or mycin plant |
| pQB-FoAPY1-R | GACAACAACC TTTGTCTTAT GGTTAC |  |
| 28a-FoAPY1-F | GGTCGCGGATCCGAATTCATGACCAAGAAGC TGACTCCCAG TC | FoAPY1 protein expressed in *Escherichia coli* |
| 28a-FoAPY1-R | GTGGTGGTGGTGCTCGAGTTA GACAACAACC TTTGTCTTAT GGTTAC |  |
| 28a-FoAPY1^PAM^-F | GGTCGCGGATCCGAATTC ATGGTGACTGCTC CTCTTGTTCT CG | PAM domain of FoAPY1 protein expressed in *Escherichia coli* |
| 28a-FoAPY1^PAM^-R | GTGGTGGTGGTGCTCGAG TTAAGCAGCACGG CCAGCAGTCT |  |
| Fo-EF1α-F | ATTGCCACACTGCCCACATT | Expression level of *EF1α* of *F. oxysporum* |
| Fo-EF1α-R | TGTCACGGACGGCGAAAC |  |
| Sl-qPCR-18S-F | GGGCATTCGTATTTCATAGTCAGA | Expression level of *18s*of tomato |
| Sl-qPCR-18S-R | GTTCTTGATTAATGAAAACATCCT |  |
